# The London Hospital

**Published:** 1893-05-20

**Authors:** 


### FESTIVAL DINNERS AND MEETINGS.

### THE LONDON HOSPITAL.

Allusion was made in our columns last week to the fact that this year the fourth quinquennial appeal in aid of the funds of this hospital is being made. A very well attended meeting, presided over by the LORD MAYOR was held in its support at the Mansion House on Thursday, May 11th. The Duke of Cambridge, the president of the hospital, once more showed the warm and kindly interest he always takes in its affairs by being present, and amongst others we noticed Lord Sandhurst, Sir John Lubbock, M.P., Colonel Fitz-George, Mr. Powell, Mr. Lidderdale, Sir Andrew Clark, Sir Mark W. Collet, Sir T. Fowell Buxton, Mr. Hale (chairman), the Bishop of Bedford, Cardinal Vaughan, Dr. Adler (the Chief Rabbi), Mr. Burdett, Dr. Stephen Mackenzie and Mrs. Mackenzie, Dr. and Mrs. Sansom, Mr. John Henry Buxton, General Sir J. Watson, and Mr. Treeves, Dr. Gilbart Smith, the Matron, Miss Lückes, and Miss R. Paget.

The LORD MAYOR spoke cordially of the pleasure it gave him to preside on this occasion, and added that no institution could be more worthy of support than the London Hospital, the largest in the country. Though not within the City bounds it was very near there, and from its position had a claim upon the support of all those connected with the City, seeing that it was universally acknowledged that there could be no more necessary site for an institution of the kind.

The Duke of CAMBRIDGE then proposed the resolution: "That the London Hospital, the largest in England, and the only large general hospital for the whole of the East End and the adjacent suburbs, is of vital importance to the working population there, and worthy of the liberal support of all classes." His Royal Higeness said it was now the third time he had addressed such a meeting on behalf of this great hospital, and he trusted that the results on this occasion would be even more successful than in past years. Some of our hospitals had lately come in for severe criticism, and he was glad that this resolution was to be seconded by one who had had such great opportunities as Lord Sandhurst as Chairman of the Select Committee of the House of Lords on Metropolitan Hospitals, of going thoroughly into the management of these institutions. He would like to read a short extract from the report of the Lords' Committee, in which is was said: "The London Hospital is an admirable hospital, doing work in a part of London where it confers inestimable benefits upon a very large and very poor population. They therefore think it is deserving of the greatest measure of charitable support." Such a recommendation as that, after the exhaustive inquiry that had been held in the working of metropolitan hospitals was a proof of the excellence of the management, and a guarantee that this institution deserved the fullest confidence and support of the public. The hospital had only £20,000 a-year which could be relied upon as annual income, and its annual expenses amounted to £60,000; it was, therefore, necessary to produce £40,000 a-year for the next five years to enable the work to be carried on. His Royal Highness then went into various particulars with regard to the number of beds (776) and the special arrangements made for Hebrew patients and children, one of the special features of the London Hospital being that it is the largest children's Hospital in London, two of the large wards being reserved for children under seven. During the past year 1,807 children were treated as in-patients. The efficiency of the institution was all that could be desired, and he trusted that every support would be given by those present and their friends outside to aid the valuable work.

Lord SANDHURST, who seconded the resolution, spoke warmly in favour of the voluntary system, saying that though he had visited hospitals in Paris, Vienna, Berlin and The Hague, he believed that nowhere would be found anything to equal the comfort and tenderness which was the rule in all our large London hospitals. He firmly held the view of the London Hospital contained in the extract from the Lords' Report just read by the Duke of Cambridge.

Sir ANDREW CLARK, as one connected for many years with the "London," cordially supported the resolution and spoke warmly in praise of the efficiency of the management. The resolution was carried unanimously.

Excellent speeches were made by the Bishop of BEDFORD, Cardinal VAUGHAN, and Dr. ADLER, Cardinal VAUGHAN specially alluding to the devotion shown by the nursing staff, and commending the efficiency of the nursing arrangements. Speeches were also made by Sir JOHN LUBBOCK, Mr. HALE, and Mr. J. H. BUXTON (the treasurer).

The meeting may on all accounts be said to have been a thoroughly satisfactory one, and it is only to be hoped that the results will be equally so, and that no difficulty will be found in raising the amount required to carry on the splendid work which is being done in the heart of the East-end by this great hospital.

